# Supplementary material for: The ClpX protease is essential for inactivating the CI master repressor and completing prophage induction in Staphylococcus aureus
Source: Nat Commun. 2023 Oct 18;14:6599. doi: 10.1038/s41467-023-42413-0 (PMC10584840; doi:10.1038/s41467-023-42413-0)
Supplement: Supplementary file 1 — Supplementary Information [file 41467_2023_42413_MOESM1_ESM.pdf]

The ClpX protease is essential for inactivating the CI master repressor and completing prophage induction in *Staphylococcus aureus*

Supplementary information

Supplementary tables

Table S1 Clp proteases in *S. aureus*

Clp protein	Locus tag (NCTC8325)	Accession number	Type
**ClpP**	SAOUHSC_00790	YP_499347	Protease
**ClpX**	SAOUHSC_01778	YP_500283	ATPase
**ClpC**	SAOUHSC_00505	YP_499078	ATPase
**ClpB**	SAOUHSC_00912	YP_499465	ATPase
**ClpL**	SAOUHSC_02862	YP_501318	ATPase
**ClpQ**	SAOUHSC_01225	YP_499761	Protease
**ClpY**	SAOUHSC_01226	YP_499762	ATPase

Table S2 Bacterial strains used in this study

Strain name	Relevant characteristics	Reference
***Escherichia coli* strains**		
BTH101	Reporter strain for BACTH; F-, *gal* E15, *gal* K16, *mcr*A1, *mcr*B1, *araD139*, *rpsL1*, *hsdR2*, *cya-99* (adenylate cyclase deficient) Str^R)	Euromedex
DC10B	*mcrA* Δ(*mrr-hsdRMS-mcrBC*) φ80*lacZ*ΔM15 Δ*lacX*74 *recA*1 *araD*139 Δ(*ara-leu*)7697 *galU galK rpsL endA1 nupG* Δ*dcm*	[1]
DH5α	*fhuA2 lac(del)U169 phoA glnV44 Φ80' lacZ(del)M15 gyrA96 recA1 relA1 endA1 thi-1 hsdR17*	Bethesda Research Laboratories
IM01B	*mcrA Δ(mrr-hsdRMS-mcrBC) φ80lacZΔM15 ΔlacX74 recA1 araD139 Δ(ara-leu)7697 galU galK rpsL endA1 nupG Δdcm ΩPhelp-hsdMS (CC1-2) ΩPN25-hsdS (CC1-1)*	[2]
JP14175	DH5α carrying pUT18c	This study
JP14176	DH5α carrying pUT18c-zip	"
JP14178	DH5α carrying pKT25-zip	"
JP14177	DH5α carrying pKT25	"
JP15397	DH5α carrying pCN41	"
JP16925	DH5α carrying pJP2668	"
JP16926	DH5α carrying pJP2669	"
JP16932	DH5α carrying pJP2601	"
JP16933	DH5α carrying pJP2603	"
JP16934	DH5α carrying pJP2602	"
JP16935	DH5α carrying pJP2604	"
JP19036	DH5α carrying pJP2578	"
JP19042	DH5α carrying pJP2584	"
JP19043	DH5α carrying pJP2585	"
JP19044	DH5α carrying pJP2586	"
JP19409	DH5α carrying pJP2589	"
JP19410	DH5α carrying pJP2590	"
JP20330	DH5α carrying pJP2597	"
JP20829	DH5α carrying pJP2596	"
JP20979	DC10B carrying pJP2605	"
JP21575	IM01B carrying pJP2638	"
JP21576	IM01B carrying pJP2642	"
JP21614	IM01B carrying pJP2636	"
JP21635	IM01B carrying pJP2632	"
JP22129	BHT101 carrying pUT18c-zip and pKT25-zip	"
JP22130	BHT101 carrying pUT18c and pKT25	"
JP22131	BHT101 carrying pJP2632 and pJP2642	"
JP22132	BHT101 carrying pJP2632 and pJP2638	"
JP22134	BHT101 carrying pJP2642 and pJP2636	"

Strain name	Relevant characteristics	Reference
JP22135	BHT101 carrying pJP2638 and pJP2636	“
Staphylococcus aureus strains		
JP1361	RN451, RN450 lysogenic for Φ11	3
JP1841	RN4220 expressing LexA_G94E	4
JP7834	RN450 Δ*clp*C (internal, partial deletion of *clp*C)	5
JP7835	RN450 Δ*clp*P (deletion of *clp*P and upstream promoter region)	6
JP7836	RN450 Δ*clp*X (internal, partial deletion of *clp*X)	“
JP7837	RN450 *clp*L::*erm*R (plasmid disruption of *clp*L)	5
JP7838	RN450 *clp*B::*erm*R (plasmid disruption of *clp*B)	“
JP7839	RN450 Δ*clp*QY (deletion of both genes)	7
JP8008	JP7835 lysogenic for 80α	This study
JP8009	JP7836 lysogenic for 80α	“
JP8010	JP7834 lysogenic for 80α	“
JP8011	JP7838 lysogenic for 80α	“
JP8012	JP7837 lysogenic for 80α	“
JP8013	JP7839 lysogenic for 80α	“
JP8106	JP7835 lysogenic for Φ11	“
JP8107	JP7836 lysogenic for Φ11	“
JP8108	JP7834 lysogenic for Φ11	“
JP8109	JP7838 lysogenic for Φ11	“
JP8110	JP7837 lysogenic for Φ11	“
JP8111	JP7839 lysogenic for Φ11	“
JP18030	RN450 Δ*clp*P	“
JP18031	RN450 Δ*clp*X	“
JP18157	RN450 Δ*clp*X, Lysogen with Φ11	“
JP18158	RN450 Δ*clp*P, Lysogen with Φ11	“
JP18169	RN450 Δ*clp*X, Lysogen with 80α	“
JP18170	RN450 Δ*clp*P, Lysogen with 80α	“
JP18269	RN450, Lysogen with Φ11	“
JP18270	RN450, Lysogen with 80α	“
JP18381	JP18157 carrying pJP2601	“
JP18382	JP18157 carrying pJP2603	“
JP18383	JP18158 carrying pJP2602	“
JP18384	JP18158 carrying pJP2604	“
JP18385	JP18157 carrying pCN51	“
JP18386	JP18158 carrying pCN51	“
JP18670	JP18269 carrying pCN51	“
JP18916	JP18270 carrying pCN51	“
JP18917	JP18169 carrying pCN51	“

Strain name	Relevant characteristics	Reference
JP18918	JP18170 carrying pCN51	“
JP18919	JP18169 carrying pJP2601	“
JP18920	JP18169 carrying pJP2603	“
JP18921	JP18170 carrying pJP2602	“
JP18922	JP18170 carrying pJP2604	“
JP19529	RN450 carrying pJP2584	“
JP19530	RN450 carrying pJP2585	“
JP19531	RN450 carrying pJP2586	“
JP19532	RN450 carrying pCN51	“
JP19533	JP18031 carrying pJP2584	“
JP19534	JP18031 carrying pJP2585	“
JP19535	JP18031 carrying pJP2586	“
JP19536	JP18031 carrying pCN51	“
JP19537	JP18030 carrying pJP2584	“
JP19538	JP18030 carrying pJP2585	“
JP19539	JP18030 carrying pJP2586	“
JP19540	JP18030 carrying pCN51	“
JP19795	RN4220 Δ*clp*P	“
JP19910	RN4220 carrying pJP2578	“
JP19911	RN4220 carrying pJP2589	“
JP19912	RN4220 carrying pJP2590	“
JP19913	RN4220 carrying pCN41	“
JP19914	JP20999 carrying pJP2578	“
JP19915	JP20999 carrying pJP2589	“
JP19916	JP20999 carrying pJP2590	“
JP19917	JP20999 carrying pCN41	“
JP19918	JP19795 carrying pJP2578	“
JP19919	JP19795 carrying pJP2589	“
JP19920	JP19795 carrying pJP2590	“
JP19921	JP19795 carrying pCN41	“
JP20045	RN450 lysogen with Φ11 ΔORF15/*ori*	“
JP20046	RN450 lysogen with 80α ΔORF20/*ori*	“
JP20189	RN450 Δ*clp*P lysogen with Φ11 ΔORF15/*ori*	“
JP20190	RN450 Δ*clp*X lysogen with 80α ΔORF20/*ori*	“
JP20191	RN450 Δ*clp*X lysogen with Φ11 ΔORF 15/*ori*	“
JP20192	RN450 Δ*clp*P lysogen with 80α ΔORF20/*ori*	“
JP20403	JP1841 carrying pCN41	“
JP20406	JP1841 carrying pJP2578	“
JP20407	JP1841 carrying pJP2590	“
JP20408	JP1841 carrying pJP2589	“

Strain name	Relevant characteristics	Reference
JP20416	JP18031 carrying pJP2605	"
JP20417	RN4220 carrying pJP2597	"
JP20418	JP1841 carrying pJP2597	"
JP20419	JP19795 carrying pJP2597	"
JP20420	JP20999 carrying pJP2597	"
JP20858	RN4220 carrying pJP2596	"
JP20859	JP1841 carrying pJP2596	"
JP20860	JP19795 carrying pJP2596	"
JP20861	JP20999 carrying pJP2596	"
JP20999	RN4220 Δ*clp*X	"
JP21189	JP18157 carrying pJP2605	"
JP21189	JP18157 carrying pJP2605	"
JP21190	JP18169 carrying pJP2605	"
JP21190	JP18169 carrying pJP2605	"
RN450	NCTC8325 cured of Φ11, Φ12 and Φ13	[3]
RN4220	Restriction-defective derivate of RN450	[8]
RN10359	RN450 lysogenic for 80α	[9]

Table S3 Plasmids used in this study

Plasmids name	Relevant characteristics	Reference
pCN41	β-lactamase reporter plasmid for *S. aureus*, Erm^R	[10]
pCN51	Cadmium-inducible expression plasmid for *S. aureus*, Erm^R	“
pMAD	Vector for efficient allelic replacement	[11]
pKT25	Bacterial Adenylate Cyclase Two-hybrid System Kit, Km^R	Euromedex
pUT18c	Bacterial Adenylate Cyclase Two-hybrid System Kit, Amp^R	Euromedex
pKT25-zip	Bacterial Adenylate Cyclase Two-hybrid System Kit positive control, Km^R	Euromedex
pUT18c-zip	Bacterial Adenylate Cyclase Two-hybrid System Kit positive control, Amp^R	Euromedex
pJP2578	pCN41 containing cI_{WT} of Φ11 cloned between SalI/BamHI	“
pJP2584	pCN51 containing cI_{WT} of Φ11 cloned between SalI/BamHI	“
pJP2585	pCN51 containing cI_{G131E} of Φ11cloned between SalI/BamHI	“
pJP2586	pCN51 containing cI_{G131*} of Φ11 cloned between SalI/BamHI	“
pJP2589	pCN41 containing cI_{G131*} of Φ11 cloned between SalI/BamHI	“
pJP2590	pCN41 containing cI_{G131E} of Φ11 cloned between SalI/BamHI	“
pJP2596	pCN41 containing *lex*A promoter cloned between SalI/BamHI	“
pJP2597	pCN41 containing *rec*A promoter cloned between SalI/BamHI	“
pJP2601	pCN51 containing *clp*X of *S. aureus* cloned between BamHI/KpnI	“
pJP2602	pCN51 containing *clp*P of *S. aureus* cloned between BamHI/KpnI	“
pJP2603	pCN51 containing *clp*X of *E. faecalis* cloned between BamHI/KpnI	“
pJP2604	pCN51 containing *clp*P of *E. faecalis* cloned between BamHI/KpnI	“
pJP2605	pCN51 containing $clpX_{I265E}$ of *S. aureus* cloned between SalI/BamHI	“
pJP2632	pKNT25 containing cI_{G131*} of Φ11 cloned between BamHI/KpnI	“
pJP2636	pKNT25 containing cI_{WT} of Φ11 cloned between BamHI/KpnI	“
pJP2638	pUT18c containing $clpX_{I265E}$ cloned between BamHI/KpnI	“
pJP2642	pUT18c containing *clp*X cloned between BamHI/KpnI	“
pJP2668	pMAD for the deletion of *clp*X	“
pJP2669	pMAD for the deletion of *clp*P	“

Table S4 Oligonucleotides used in this study

Plasmid name	Oligo name	Sequence (5'-3')
**Sequencing**		
pMAD	pMAD_F	GTCCCAATATAATCATTTATCAACTCTTTTAC
	pMAD_R	GAAGAATCATAATGGGGAAGGCC
pCN51	pCN51_F	GGTGGTCAACTTTAGAAAAGAAGG
	pCN51_R	GATATCAAAATTATACATGTCAACGATAATAC
pCN41	pCN41_F	GGATAACCGTATTACCGCCTTTG
	pCN41_R	CTCTTTGGCATGTGAACTGTTTG
pKT25	pKT25-F	GCAGTTCGGTGACCAGC
	pKT25-R	ATGTGCTGCAAGGCGATTAAG
pUT18c	pUT18c-F	GATGTACTGGAAACGGTGCC
	pUT18c-R	CTTAACTATGCGGCATCAGAGC
**Mutant verification**		
Δ*clp*P	ClpP-9m	GGAGAAATGGTATCAACTGG
	ClpP-10c	TCTGGTCAACAATGGTATTC
Δ*clp*X	ClpX-8c	CAATTGTATCGTCTGGGTCC
	ClpX-7m	GAATTAGCCGGTAAAGAAGC
**Southern blotting probes**		
Φ11 probe	Probe 11_F	ATGCAAGACCAATCATTAAAATTAGTAAAAC
	Probe 11_R	GATAAGCGTGGTTATATTAAGAAGTGAATGTTAC
80α probe	Probe 80a_F	GTAACAGTATCAAACACTTAAGAAAAAATTC
	Probe 80a_R	CATAGTGACCTCCTACCATCTCATG
**Plasmid construction**		
pJP2668	ClpX-16cS	ACGCGTCGACAATTTTGTCTTCTTTAGTGC
	ClpX-5m	CTCTTTCTGCGGAAAAGACGAACTATACGACGCAGAAGG
	ClpX-4c	GTCTTTTCCGCAGAAAGAGC
	ClpX-13mB	CGCGGATCCAAATTTAAAGAAGTCCCAGA
pJP2669	ClpP-8cB	ATATCGGATCCAACTGCACCTATACCTGAACG
	ClpP-7m	CGTGCATATGATATATACTCAGCTTAATTG
	ClpP-6c	TGAGTATATATCATATGCACG
	ClpP-5mS	GGTACCCGGGAGCTGGAAAGTTTAATGAAGG
pJP2638	ClpX_152_F_B	CTAGAGGATCCAATGTTTAAATTCAATGAAGATGAAGAAAATTTG
	ClpX_154_R_K	AGCTCGGTACCGAGCTTTTCACTTTTATAACACATCAATGA
pJP2642	ClpX_152_F_B	CTAGAGGATCCAATGTTTAAATTCAATGAAGATGAAGAAAATTTG
	ClpX_156_R_K	AGCTCGGTACCGAGCTTTTCACTTTTATAACACATCAATGA

Plasmid name	Oligo name	Sequence (5'-3')
pJP2636	C1_148_F_B	CTAGAGGATCCAATGGATAAAAAAGAATTAGCGAAATTTATAG
	C1_151_R_K	AGCTCGGTACCTGCAATACAACTTTGCCCATTACTTTAATATT
pJP2632	NTD_144_F_B	CTAGAGGATCCAATGGATAAAAAAGAATTAGCGAAATTTATAG
	NTD_147_R_K	CGAGCTCGGTACCTGAGCACCAGTTGCACCAC
pJP2578	WT CI_F_46_S	TGCAGGTCGACTCACAATACAACTTTGCCCATTACTTTAATATTAC
	Delta Cro_54_R_B	CCCGGGGATCCTTCTCAACTTTATTAAATTCCATTGCATG
pJP2589	MT_Delta Cro_54_R_B	CCCGGGGATCCTTCTCAACTTTATTAAATTCCATTGCATG
	MT_G131STOP_53_F	CTGCAGGTCGACTTAAGCACCAGTTGCACCAC
pJP2590	MT_Delta Cro_54_R_B	CCCGGGGATCCTTCTCAACTTTATTAAATTCCATTGCATG
	WT CI_F_46_S	TGCAGGTCGACTCACAATACAACTTTGCCCATTACTTTAATATTAC
pJP2596	LexA IG_F_S	CCTGCAGGTCGACGTTAATTCTCTCATATATAGGCACTCCC
	LexA IG_R_B	CCGGGGATCCATTTATTGTAAAACATCATTTTCACTCCTAGAAC
pJP2597	RecA IG_F_S	GCAGGTCGACGCTTAGAACAACAAATTAATTGTATTATCGATAAAAAT
	RecA IG_R_B_102	ACCCGGGGATCCGTTGATGTAGTTGA AACTCGGC
pJP2584	Phi11 C1_63_F S	TGCAGGTCGACGTAAATTTAAGGAGGTAAGAAAATGGATAAAAAAGAATTAG
	Phi11 C1_64_R_B	CCCGGGGATCCTCACAATACAACTTTGCCCATTACTTTAATATTAC
pJP2586	Phi11 C1_63_F S	TGCAGGTCGACGTAAATTTAAGGAGGTAAGAAAATGGATAAAAAAGAATTAG
	Phi11 G131*_65 RB	CCCGGGGATCCTCAAGCACCAGTTGCACCAC
pJP2585	Phi11 C1_63_F S	TGCAGGTCGACGTAAATTTAAGGAGGTAAGAAAATGGATAAAAAAGAATT AG
	Phi11 C1_64_R_B	CCCGGGGATCCTCACAATACAACTTTGCCCATTACTTTAATATTAC
pJP2601	ClpX_85_F_S	CTGCAGGTCGACCAATCTAGTATAGTCTTTAACGAATAGGGG
	ClpX_86_R_B	ACCCGGGGATCCGAGCTTTTCACTTTTATAACACATCAATG
pJP2603	ClpX_106_F_B	TCTAGAGGATCCATGTACGACAATACGGATAATAACG
	ClpX_107_R_K	TTACCGGTACCTTATACAGCTTCTTTATCTTTTTTGTTATAAA
pJP2602	ClpP_110_F_B	CTAGAGGATCCTATGTAAAATAATGAGTAACAGTTATTACAAGGAGG
	ClpP_111_R_K	TTATTTTGTTTCAGGTACCATCACTTC
pJP2604	ClpP_108_F_B	CTAGAGGATCCTAGGCATTCAAAGTGCTTTGTGATAG

Plasmid name	Oligo name	Sequence (5'-3')
	ClpP_109_R_K	GCTCGGTACCTTTTTAATTTAAGGCGCTACTATTTTC CATTAC
pJP2605	ClpX_85_F_S	CTGCAGGTCGACCAATCTAGTATAGT CTTTAACGAATAGGGG
	I265E_70_R	AGAAACCCTCAACTTTTTCACCAA GACGGCGCTTAAT
	I265E_72_F	GTGAAAAAGTTGAGGGTTTCTCAAG CAATGAAGCTGATAAATATG
	ClpX_86_R_B	ACCCGGGGATCCGAGCTTTTCACTTT TATAACACATCAATG
**Prophage verification**		
Φ11	Sa5-F	AAAGATGCCAAACTAGCTG
	Sa5-R	CTTGTGGTTTTGTTCTGG
80α	Sa7-F	GTCCGGTAGCTAGAGGTC
	Sa7-R	GGCGTATGCTTGACTGTGT

Supplementary Figures and Figure Legends

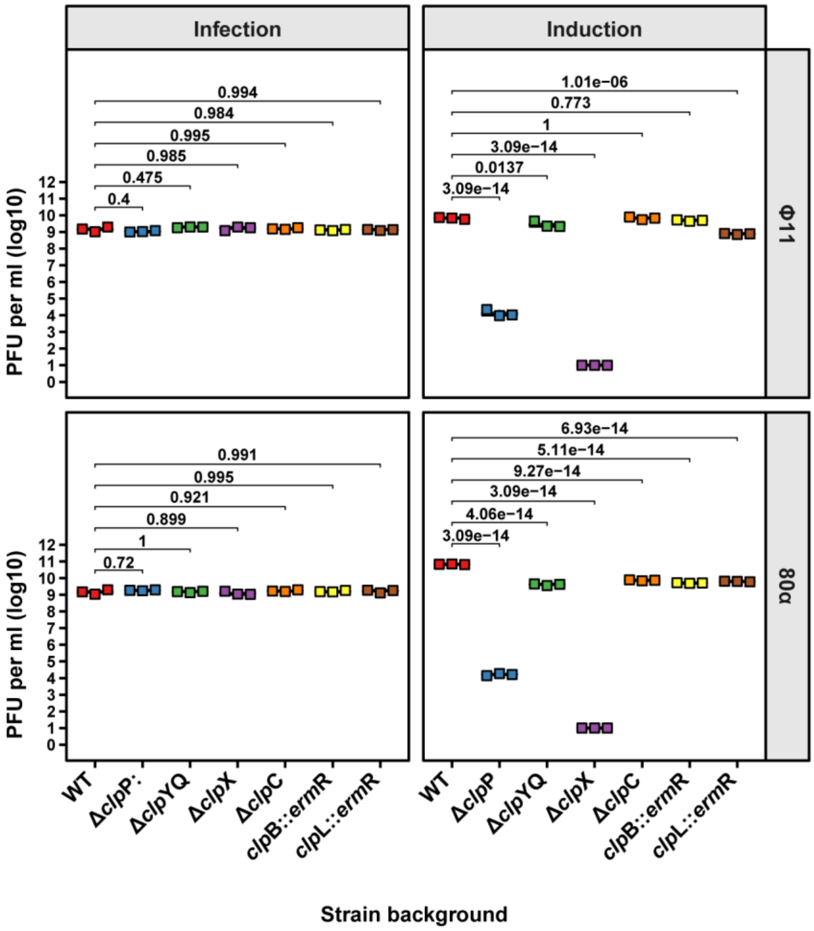

Fig. S1. ClpP and ClpX are involved in phage induction but not phage infection. The defined RN450 derivative mutant strains were either infected with the indicated phages or lysogenic derivatives induced by MC addition. Plaque formation was assessed on a lawn of RN4220. Bold horizontal lines in each boxplot represent the median and lower and upper hinges the first and third quartiles, respectively (n=3 biological replicates). Assessment of statistically significant differences between groups was performed using ANOVA followed by Tukey's HSD post-test. p-values are indicated above each comparison.

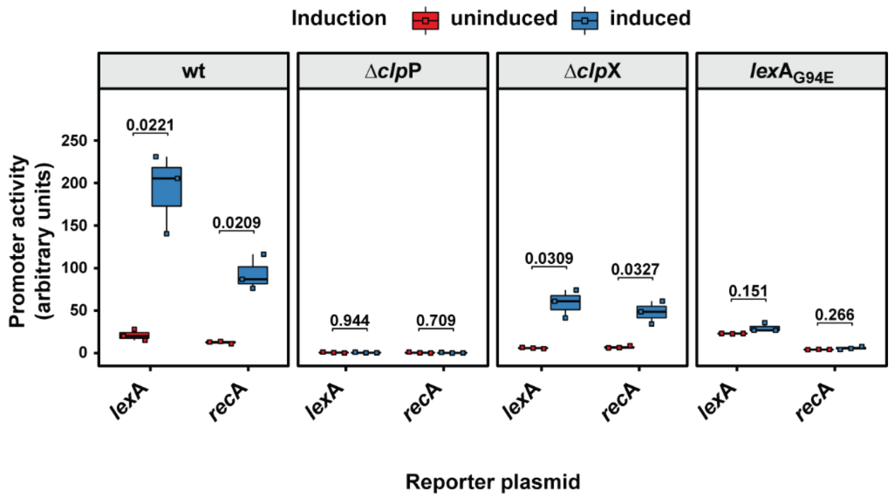

Fig. S2. Distinct roles for ClpP and ClpX in SOS response induction. Reporter plasmids were designed to place the β-lactamase reporter gene (*bla*Z) of plasmid pCN41 under the control of the SOS-controlled promoters of *lex*A or *rec*A. RN4220 derivative strains containing the indicated plasmids were grown to exponential phase, split and the SOS response induced in one half of the culture with MitC (blue boxplots) while the other half was left untreated (red boxplots). Samples were taken 90 min after induction. Bold horizontal lines in each boxplot represent the median and lower and upper hinges the first and third quartiles, respectively (n=3 biological replicates). Assessment of statistically significant differences between groups was performed using a two-sided Student's t-test. p-values are indicated above each comparison.

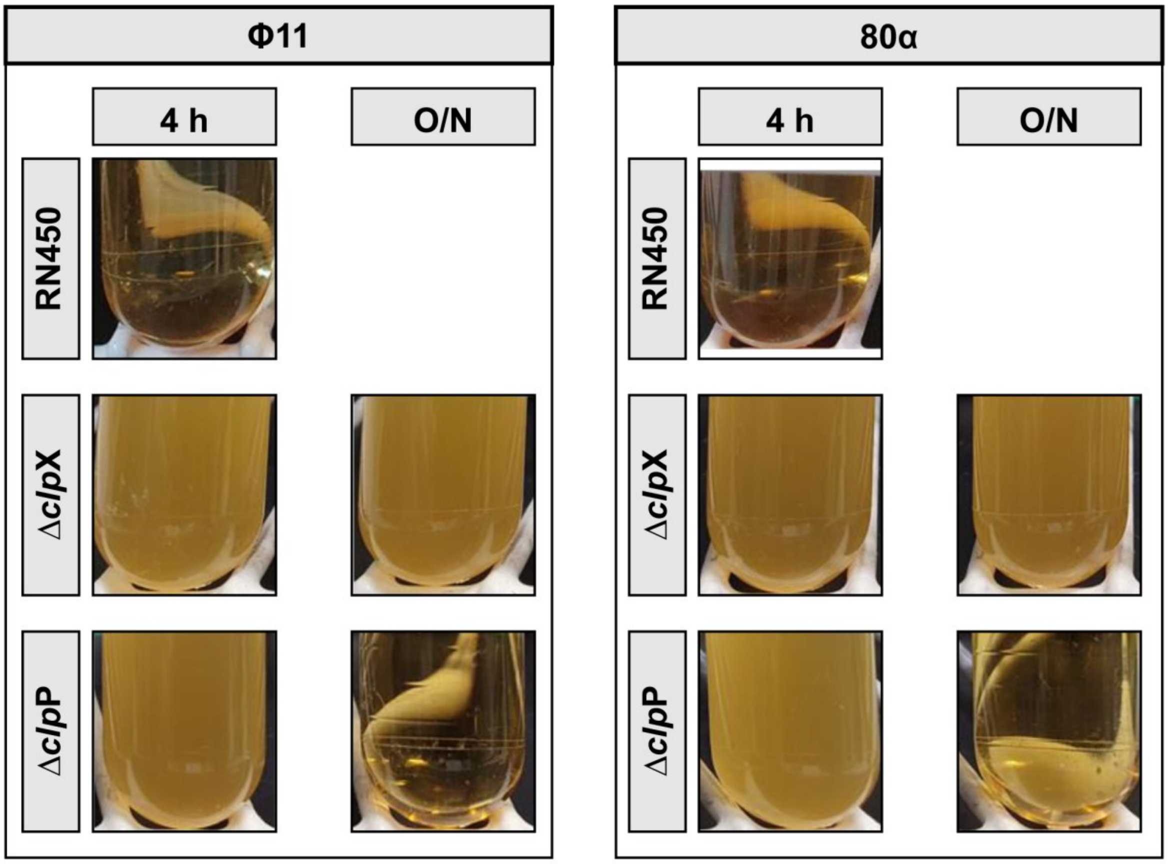

Fig. S3. Lysis behaviour of clpX and clpP mutans. The indicated RN450 derivatives lysogenic for either Φ11 or 80α were grown to exponential phase followed by mitomycin C induction of the lytic phage cycle. The cultures were incubated 4 h at 30 ℃, 80 rpm followed by an overnight incubation (O/N) on the bench. Representative images of cell lysis at different time points are shown.

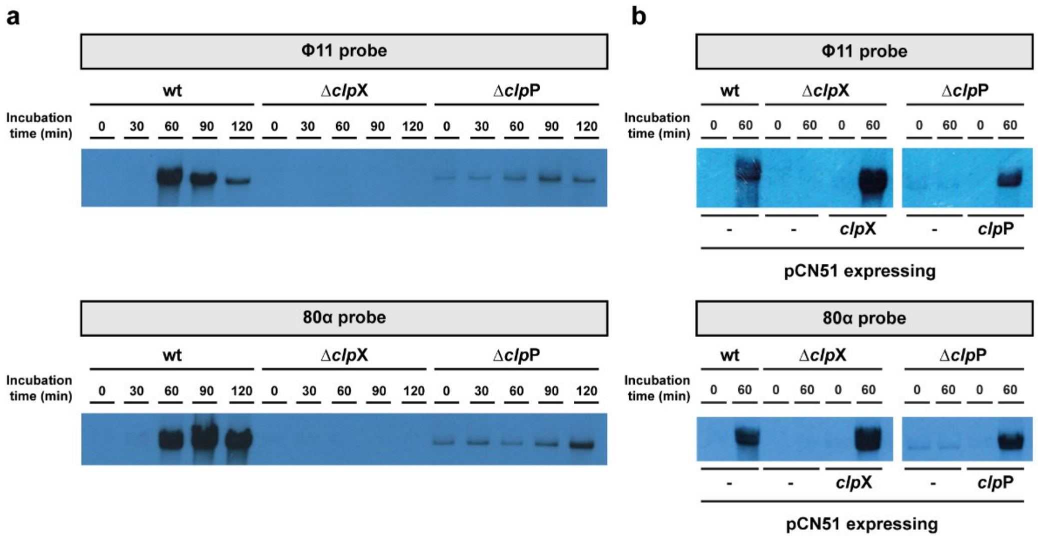

Fig. S4. ClpP and ClpX impact phage replication. (a) The defined RN450 derivative strains lysogenic for either Φ11 (wt RN451, Δ*clp*X JP18031, Δ*clp*P JP18030) or 80α (wt JP18270, Δ*clp*X JP18169, Δ*clp*P JP18170) were induced by MitC addition and samples for DNA extraction were taken at the time points indicated. Crude DNA lysates for Southern-blotting analysis were then separated by agarose gel electrophoresis, transferred onto a nitrocellulose membrane, and replicating phage DNA visualized using a phage-specific DIG-labelled DNA probe. **(b)** The *clp*X and *clp*P genes were cloned into the cadmium-inducible expression plasmid pCN51 (pJP2601 and pJP2602, respectively), introduced into the defined strains and induced by MitC addition. Samples were taken at the defined timepoints for Southern blotting analysis as described in **(a)**. Expression from the pCN51 plasmids was maintained throughout the experiment by the addition of 1 μM $CdCl_2$.

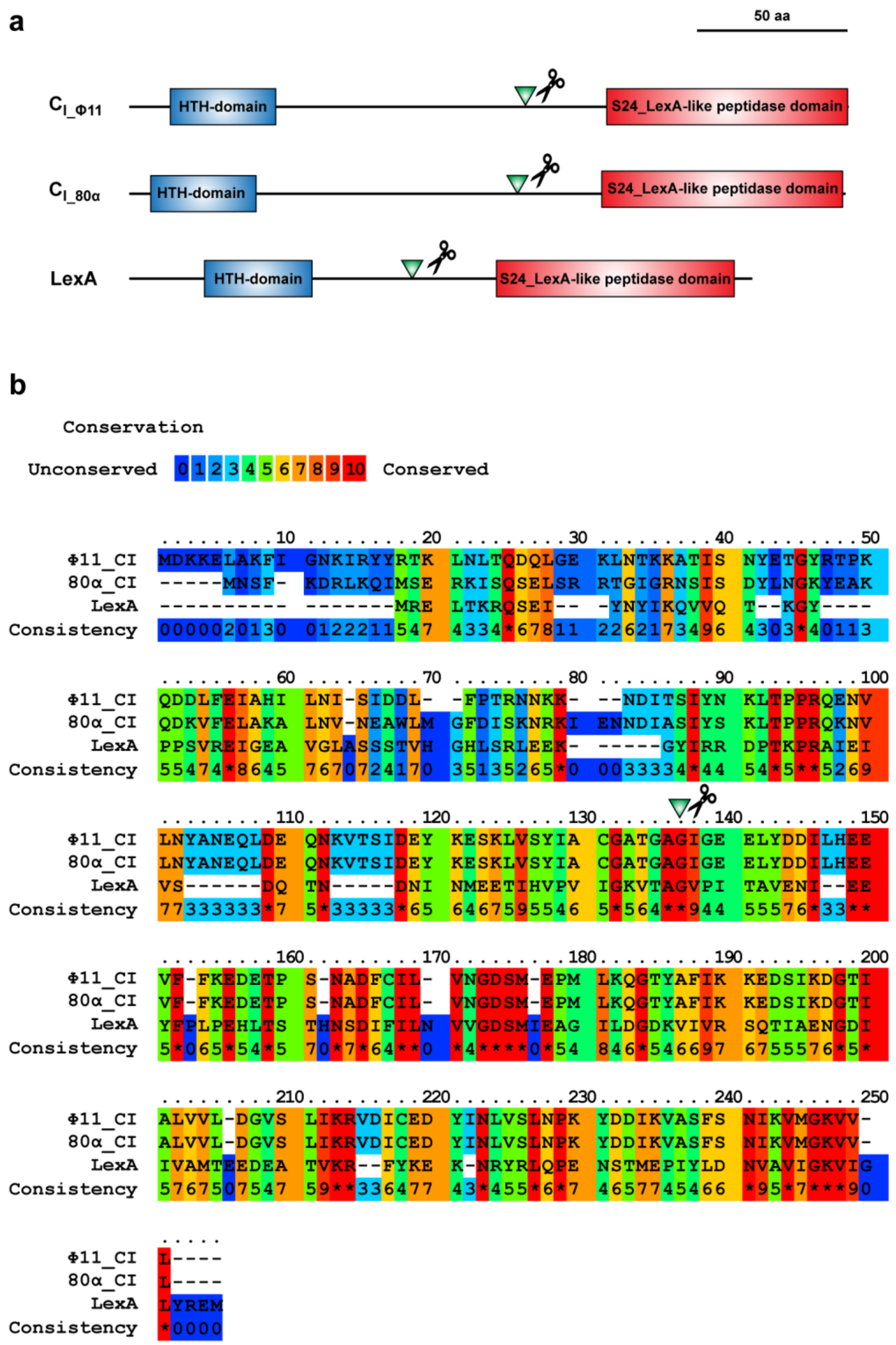

Fig. S5. Comparison of phage repressors and LexA proteins. (A) Schematic representation of the domain architecture of the SOS master regulator LexA with the CI repressor proteins of Φ11 and 80α. **(B)** The three proteins were aligned, and conservation of residues compared using PRALINE web server (https://www.ibi.vu.nl/programs/pralinewww/).

A green triangle accompanied by a scissors icon indicates the conserved glycine residue where cleavage occurs during RecA*-mediated autocleavage.

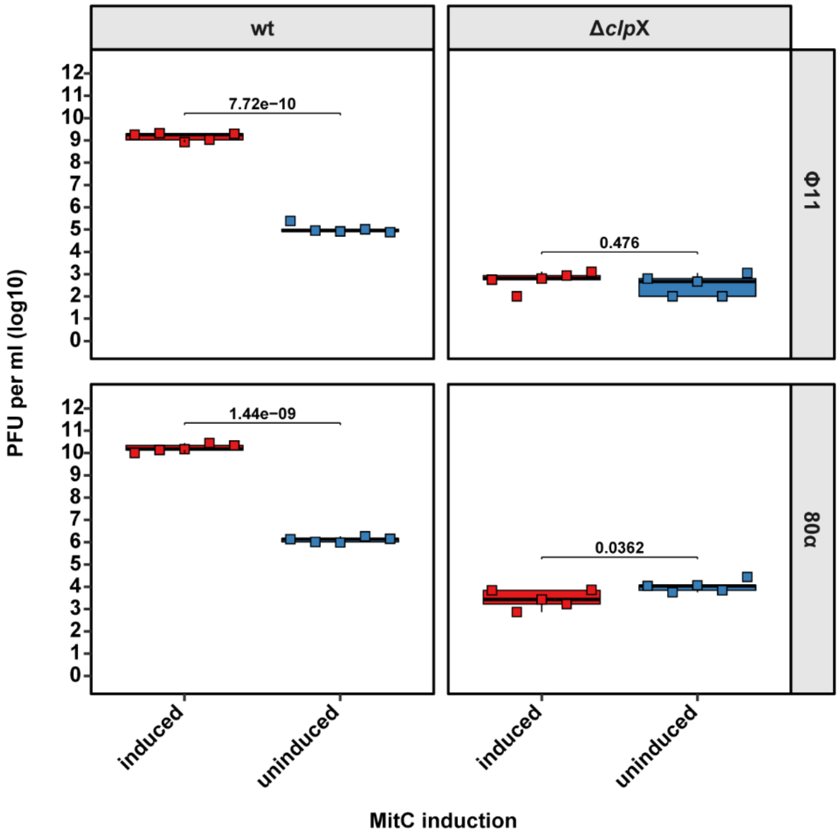

Fig. S6. ClpX is essential for SOS-mediated prophage induction. The indicated RN450 prophage lysogens were grown to exponential phase and either induced with MitC or left untreated to monitor spontaneous prophage induction. Plaque formation was assessed on a lawn of RN4220. Bold horizontal lines in each boxplot represent the median and lower and upper hinges the first and third quartiles, respectively (n=3 biological replicates). Assessment of statistically significant differences between groups was performed using a two-sided Student's t-test on $\log_{10}$ transformed data. p-values are indicated above the respective comparison.

Supplementary References

1. Monk IR, Shah IM, Xu M, Tan MW, Foster TJ. Transforming the untransformable: application of direct transformation to manipulate genetically *Staphylococcus aureus* and *Staphylococcus epidermidis*. *mBio* **3**, (2012).

2. Monk IR, Tree JJ, Howden BP, Stinear TP, Foster TJ. Complete Bypass of Restriction Systems for Major *Staphylococcus aureus* Lineages. *mBio* **6**, e00308-00315 (2015).

3. Novick R. Properties of a cryptic high-frequency transducing phage in *Staphylococcus aureus*. *Virology* **33**, 155-166 (1967).

4. Maiques E*, et al.* β-lactam antibiotics induce the SOS response and horizontal transfer of virulence factors in *Staphylococcus aureus*. *J Bacteriol* **188**, 2726-2729 (2006).

5. Frees D*, et al.* Clp ATPases are required for stress tolerance, intracellular replication and biofilm formation in *Staphylococcus aureus*. *Mol Microbiol* **54**, 1445-1462 (2004).

6. Frees D, Qazi SN, Hill PJ, Ingmer H. Alternative roles of ClpX and ClpP in *Staphylococcus aureus* stress tolerance and virulence. *Mol Microbiol* **48**, 1565-1578 (2003).

7. Frees D, Thomsen LE, Ingmer H. *Staphylococcus aureus* ClpYQ plays a minor role in stress survival. *Arch Microbiol* **183**, 286-291 (2005).

8. Kreiswirth BN*, et al.* The toxic shock syndrome exotoxin structural gene is not detectably transmitted by a prophage. *Nature* **305**, 709-712 (1983).

9. Ubeda C, Barry P, Penades JR, Novick RP. A pathogenicity island replicon in *Staphylococcus aureus* replicates as an unstable plasmid. *Proc Natl Acad Sci U S A* **104**, 14182-14188 (2007).

10. Charpentier E, Anton AI, Barry P, Alfonso B, Fang Y, Novick RP. Novel cassette-based shuttle vector system for Gram-positive bacteria. *Appl Environ Microbiol* **70**, 6076-6085 (2004).

11. Arnaud M, Chastanet A, Debarbouille M. New vector for efficient allelic replacement in naturally nontransformable, low-GC-content, gram-positive bacteria. *Appl Environ Microbiol* **70**, 6887-6891 (2004).
