# Supplementary material for: The ClpX protease is essential for inactivating the CI master repressor and completing prophage induction in Staphylococcus aureus
Source: Nat Commun. 2023 Oct 18;14:6599. doi: 10.1038/s41467-023-42413-0 (PMC10584840; doi:10.1038/s41467-023-42413-0)
Supplement: Supplementary file 3 — Description of additional supplementary files [file 41467_2023_42413_MOESM3_ESM.docx]

**Description of additional supplementary files**

Title: Supplementary software

Description:

This file contains a collection of R scripts that were used to map various attachment sites for phages 80α and Ф11. attB indicates scripts used to map unoccupied chromosomal attachment sites, attL and attR phage-occupied left and right attachment sites, respectively, and attP phage attachment sites representing circular, excised phages.
