# Supplementary material for: The ClpX protease is essential for inactivating the CI master repressor and completing prophage induction in Staphylococcus aureus
Source: Nat Commun. 2023 Oct 18;14:6599. doi: 10.1038/s41467-023-42413-0 (PMC10584840; doi:10.1038/s41467-023-42413-0)
Supplement: Supplementary file 5 — Reporting Summary [file 41467_2023_42413_MOESM5_ESM.pdf]

nature portfolio

Corresponding author(s): Andreas F Haag

Last updated by author(s): Sep 29, 2023

Reporting Summary

Nature Portfolio wishes to improve the reproducibility of the work that we publish. This form provides structure for consistency and transparency in reporting. For further information on Nature Portfolio policies, see our Editorial Policies and the Editorial Policy Checklist.

Statistics

For all statistical analyses, confirm that the following items are present in the figure legend, table legend, main text, or Methods section.

n/a	Confirmed	
☐	☒	The exact sample size (*n*) for each experimental group/condition, given as a discrete number and unit of measurement
☐	☒	A statement on whether measurements were taken from distinct samples or whether the same sample was measured repeatedly
☐	☒	The statistical test(s) used AND whether they are one- or two-sided<br>*Only common tests should be described solely by name; describe more complex techniques in the Methods section.*
☐	☒	A description of all covariates tested
☐	☒	A description of any assumptions or corrections, such as tests of normality and adjustment for multiple comparisons
☐	☒	A full description of the statistical parameters including central tendency (e.g. means) or other basic estimates (e.g. regression coefficient) AND variation (e.g. standard deviation) or associated estimates of uncertainty (e.g. confidence intervals)
☐	☒	For null hypothesis testing, the test statistic (e.g. *F*, *t*, *r*) with confidence intervals, effect sizes, degrees of freedom and *P* value noted<br>*Give P values as exact values whenever suitable.*
☒	☐	For Bayesian analysis, information on the choice of priors and Markov chain Monte Carlo settings
☒	☐	For hierarchical and complex designs, identification of the appropriate level for tests and full reporting of outcomes
☒	☐	Estimates of effect sizes (e.g. Cohen's *d*, Pearson's *r*), indicating how they were calculated

Our web collection on statistics for biologists contains articles on many of the points above.

Software and code

Policy information about availability of computer code

Data collection	No software was used
Data analysis	Preparation of custom reference genomes Genomic DNA of the RN450 (NCTC8325-4) reference stock in the lab (JP1250) was extracted and sequenced as described above. Next, reads were assembled to a scaffold of the deposited NCTC8325 (GenBank Accession CP000253 [https://www.ncbi.nlm.nih.gov/nuccore/87201381]) reference genome using the PATRIC Bioinformatics Resource Center55. The three prophages of NCTC8325 were deleted and any mutations identified were curated manually. Sequencing reads were then reassembled to the curated genome (GenBank Accession CP097113 [https://www.ncbi.nlm.nih.gov/nuccore/CP097113]) as described above for verification. The sequences of either Φ11 (RefSeq Accession NC_004615 [https://www.ncbi.nlm.nih.gov/nuccore/NC_004615]) or 80α (RefSeq Accession NC_009526 [https://www.ncbi.nlm.nih.gov/nuccore/NC_009526]) were inserted into attachment sites 5 and 749, respectively and correct insertion verified by assembly of genome sequencing reads for strain JP18269 (GenBank Accession CP097114) or JP18270 (GenBank Accession CP097115) for Φ11 or 80α lysogens, respectively. The curated genomes were next uploaded to the Galaxy web platform, and we used the public server at usegalaxy.org to analyse the data56. Genomes were reannotated using Prokka v1.14.657, 58 (Galaxy Version 1.14.6+galaxy1). Analysis of whole genome sequencing data. The sequencing data were uploaded to the Galaxy web platform, and we used the public server at usegalaxy.org to analyse the data56. The read quality of paired reads was assessed using FastQC v0.11.859 (Galaxy Version 0.72+galaxy1) followed by adapter trimming using Trimmomatic v0.3860 (Galaxy Version 0.38.0) and standard setting for paired end reads and Illumina data. Trimmed reads were then reassessed using FastQC and mapped to custom genomes of RN450 reference genome containing either prophage Φ11 or 80α using the Burrows-Wheeler Alignment Tool v0.7.17.461, 62 (Galaxy Version 0.7.17.4) with default settings and saved as BAM files. To normalize sequence coverage across experiments, we first filtered the aligned reads mapping to the bacterial chromosome and not belonging to the prophage using the BAMTools v2.4.0 Filter tool63 (Galaxy Version 2.4.1). The number of mapped reads for each experiment were extracted from the filtered BAM files using the SAMTools stats utility v1.964 (Galaxy Version 2.0.2+galaxy2). Average genome coverage was calculated using the following formula: average genome coverage = (number of mapped reads) × (average read length (bp)) / (genome

length (bp)). Next, we computed the relative coverage over 50 bp sliding windows along the entire chromosome without normalization for each of the experiments of the unfiltered BAM files using the bamCoverage tool of the deepTools2 package v3.3.265 (Galaxy Version 3.3.2.0.0). These coverage files were saved in bedgraph format and further analysed using RStudio v2021.9.1.372 (Ghost Orchid)66 and R v4.1.267. Samples were normalised by dividing each coverage window by the average genome coverage calculated for each experiment. Final coverage graphs were plotted in RStudio using ggplot2 v3.3.568 and genome organization around the plotted area extracted from the gff3 file produced by the Prokka annotation and graphed using gggplot2 and the gggenes package v0.4.169.

Determination of prophage integration and excision frequency. Reads were mapped onto either the prophage-free, prophage containing or circularised phage genomes to using BWA61, 62 to determine read coverage of the bacterial attB, the prophage left attachment site attL and the excised phage attP sites, respectively. Reads around the relevant attachment site (+- 800 bp), which had a matching mate read were extracted using the Samtools view64 command in Galaxy. The reduced dataset was then further filtered in Rstudio (for filtering scripts, see supplementary material) and only reads in which the paired reads mapped across the attachment site were counted. For a pair to be counted as overlapping, either both reads needed to map clearly to the two different sides of the attachment side or, if the reads contained the attachment side, they needed to begin 10 bp before its start to be considered. Reads were normalised by dividing the reads for each att site by the average genome coverage calculated for each experiment. The fraction of integrated prophage was calculated by dividing the reads mapping to attL by the sum of the reads mapping to both attL and attB. The fraction of circularised and excised phage was calculated by dividing the reads mapping to attP by the sum of the reads mapping to attP and attL.

Statistical analyses. Statistical analysis was performed as indicated in the figure legend. In general, phage titres were log10-transformed and analysed by either One-Way ANOVA followed by Tukey's HSD post-test or using a Student's unpaired two-tailed t-test as appropriate for the relevant comparison. Promoter activity data were analysed on raw activity data by either One-Way ANOVA followed by Tukey's HSD post-test and Bonferroni correction or using a Student's unpaired two-tailed t-test as appropriate for the relevant comparison. All analysis was done using RStudio.

For manuscripts utilizing custom algorithms or software that are central to the research but not yet described in published literature, software must be made available to editors and reviewers. We strongly encourage code deposition in a community repository (e.g. GitHub). See the Nature Portfolio guidelines for submitting code & software for further information.

Data

Policy information about availability of data

All manuscripts must include a data availability statement. This statement should provide the following information, where applicable:

- Accession codes, unique identifiers, or web links for publicly available datasets
- A description of any restrictions on data availability
- For clinical datasets or third party data, please ensure that the statement adheres to our policy

All underlying data are provided within the manuscript and raw data as well as statistical analyses performed are provided as supplementary source data files. R Scripts for analysis are provided in the Supplementary Software file. Reference sequences and whole genome sequencing reads can be accessed through Bioproject (https://www.ncbi.nlm.nih.gov/bioproject/) PRJNA835099.

Research involving human participants, their data, or biological material

Policy information about studies with human participants or human data. See also policy information about sex, gender (identity/presentation), and sexual orientation and race, ethnicity and racism.

Reporting on sex and gender	N/A
Reporting on race, ethnicity, or other socially relevant groupings	N/A
Population characteristics	N/A
Recruitment	N/A
Ethics oversight	N/A

Note that full information on the approval of the study protocol must also be provided in the manuscript.

Field-specific reporting

Please select the one below that is the best fit for your research. If you are not sure, read the appropriate sections before making your selection.

[x] Life sciences [ ] Behavioural & social sciences [ ] Ecological, evolutionary & environmental sciences

For a reference copy of the document with all sections, see nature.com/documents/nr-reporting-summary-flat.pdf

Life sciences study design

All studies must disclose on these points even when the disclosure is negative.

Sample size	No sample size calculation was performed. Three independent biological replicates were generally considered to provide sufficient evidence for observed phenotypes.

Data exclusions	No data were excluded.
Replication	All experiments were performed at least in three independent biological replicates (all successful) with the exception of the whole genome sequencing experiment, which was performed only once. Whole genome sequencing was performed only once as Southern blots served as independent controls for the observations made. These were performed at least three times successfully and confirmed sequencing observations.
Randomization	Not relevant for this study. Individual experiments contained internal control (i.e. wt strains, empty plasmid control) as well as test strains (i.e. mutants). Comparisons were made between defined test strains/conditions and no other selection was applicable. For each experimental comparison, at least three independent biological repeats were performed (as indicated above). Biological repeats were highly reproducible.
Blinding	See randomization.

Reporting for specific materials, systems and methods

We require information from authors about some types of materials, experimental systems and methods used in many studies. Here, indicate whether each material, system or method listed is relevant to your study. If you are not sure if a list item applies to your research, read the appropriate section before selecting a response.

Materials & experimental systems

n/a	Involved in the study
☒	☐ Antibodies
☒	☐ Eukaryotic cell lines
☒	☐ Palaeontology and archaeology
☒	☐ Animals and other organisms
☒	☐ Clinical data
☒	☐ Dual use research of concern
☒	☐ Plants

Methods

n/a	Involved in the study
☒	☐ ChIP-seq
☒	☐ Flow cytometry
☒	☐ MRI-based neuroimaging
